# 3D printed spinning cup-shaped device for immunoaffinity solid-phase extraction of diclofenac in wastewaters

**DOI:** 10.1007/s00604-022-05267-9

**Published:** 2022-04-02

**Authors:** Enrique Javier Carrasco-Correa, José Manuel Herrero-Martínez, Ernesto Francisco Simó-Alfonso, Dietmar Knopp, Manuel Miró

**Affiliations:** 1 CLECEM group, Department of Analytical Chemistry, University of Valencia, University of Valencia, C/Doctor Moliner 50, 46100 Burjassot Valencia, Spain; 2 Department of Chemistry, Technical University of Munich, Elisabeth-Winterhalter Str. 6, 83177 München, Germany; 3 FI-TRACE Group, Department of Chemistry, University of Balearic Islands, Carretera de Valldemossa, km 7.5, 07122 Palma de Mallorca, Spain

**Keywords:** 3D printing, Immunosorbent, Extraction device, Diclofenac, Wastewater

## Abstract

This article reports current research efforts towards designing bespoke microscale extraction approaches exploiting the versatility of 3D printing for fast prototyping of novel geometries of sorptive devices. This is demonstrated via the so-called 3D printed spinning cup-based platform for immunoextraction of emerging contaminants using diclofenac as a model analyte. A new format of rotating cylindrical scaffold (containing a semispherical upper cavity) with enhanced coverage of biorecognition elements, and providing elevated enhancement factors with no need of eluate processing as compared with other microextraction stirring units is proposed. Two distinct synthetic routes capitalized upon modification of the acrylate surface of stereolithographic 3D printed parts with hexamethylenediamine or branched polyethyleneimine chemistries were assayed for covalent binding of monoclonal diclofenac antibody.

Under the optimized experimental conditions, a LOD of 108 ng L^−1^ diclofenac, dynamic linear range of 0.4–1,500 µg L^–1^, and enrichment factors > 83 (for near-exhaustive extraction) were obtained using liquid chromatography coupled with UV–Vis detection. The feasibility of the antibody-laden device for handling of complex samples was demonstrated with the analysis of raw influent wastewaters with relative recoveries ranging from 102 to 109%. By exploiting stereolithographic 3D printing, up to 36 midget devices were fabricated in a single run with an estimated cost of mere 0.68 euros per 3D print and up to 16 €/device after the incorporation of the monoclonal antibody.

**Graphical abstract:**

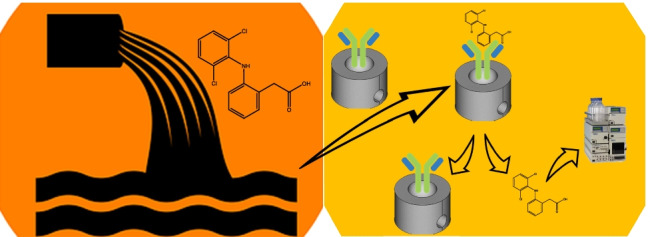

**Supplementary Information:**

The online version contains supplementary material available at 10.1007/s00604-022-05267-9.

## Introduction

3D printing (additive manufacturing) has landed in the analytical chemistry field to revolutionize the way that analytical methodologies are conceived. This dawn over the last few years is exemplified by a plethora of review articles on 3D printed platforms and (sensing) devices in the (bio)analytical field [1–3]. 3D printing can be performed exploiting a variety of cost-effective technologies, such as fused deposition modelling (FDM) and stereolithography (SLA) among others [1, 4]. However, SLA offers a series of advantages for the development of breakthrough analytical applications compared to their counterparts, such as (i) smoother prints, (ii) appropriate resolution in all the axes, (iii) acceptable organic solvent compatibility and (iv) sufficient tightness to the flowing of solutions/solvents at moderate/high pressure [4]. SLA can be breakdown in several stereolithographic sub-modes but low force stereolithography (LFS), based on the point-by-point polymerization of every polymer layer by irradiation with a laser is the common choice for analytical applications. Although other alternatives such as digital light processing or masked stereolithography feature similar resolution, their analytical potential is usually jeopardized by the larger amount of non-polymerized residues. In any case, the unique features of LFS (good resolution and tightness, low porosity and fast polymerization) makes this technology most amenable to applications in analytical science [5, 6]. In fact, bespoke 3D printed objects that enable stand out from standard devices and geometries can be designed and incorporated at will in any step of the analytical process, including sample preparation. Here, a gold standard procedure, *namely*, solid-phase extraction (SPE), is strongly associated with cartridges that have dominated the SPE formats, although other supports, such as stirring devices, have been exploited yet to a less extent. In fact, stir-bar sorptive extraction (SBSE) [7] thanks to its simplicity, robustness and high extraction efficiency and capacity has attracted the interest of the (bio)analytical chemistry community for efficient sample preparation, yet its coupling with 3D SLA printing exploiting material properties of the photopolymerized resin has not been described as of yet.

SBSE is commonly composed of a polydimethylsiloxane film, coated onto a glass jacket with an incorporated magnet core, in a cylindrical or disk shape [7–9]. The scientific community has recently introduced novel SBSE coatings to exploit more selective materials, such as molecularly imprinted polymers [10, 11], metal organic frameworks [12, 13], carbonaceous materials [14, 15] and polymer monoliths [16]. However, the use of highly selective biomaterials such as antibodies (Abs) [17] or aptamers [18, 19] has been only occasionally used.

The main problem of SBSE for obtaining high enrichment factors is the need of a large volume of eluent to cover the stir-bar-coated material entirely. In order to overcome this issue, many authors have chosen to combine SBSE with head-space gas chromatography or thermal desorption so as to avoid the use of solvents in the elution step [20, 21]. Nonetheless, this approach is restricted to volatile compounds and other techniques, such as liquid chromatography or electrophoresis, are shunned. Another option is to evaporate and reconstitute the eluate, but this is a non-sustainable, and cost-energetic procedure that lacks green credentials. Therefore, there is a quest of developing novel miniaturized platforms for simplification of sample treatment workflows while enabling elution with the minimum amount of solvent possible.

In this sense, the use of 3D printing allows the fabrication of midget devices with dedicated features that can be adapted to any methodology in SBSE. For example, Šrámková et al*.* [22] used an FDM printer to fabricate a 3D printed cage-shaped holder to contain external polymer micro- and nanofibres to extract bisphenols from waters. In this case, the authors selected polycaprolactone fibres as sorbent, while the 3D printed cage was used only as a magnet-containing holder. Again, the main shortcoming of this device is the still large volume needed for the elution step (5 mL), which jeopardizes the preconcentration capacity (only up to about 10 times). Also, the use of FDM technology led to more porous materials than those of LFS and could led to unwanted extraction by the support material itself. Hence, it is necessary to create tailor-made devices to increase the preconcentration efficiency while minimizing the non-selective extraction of the pristine-printed material.

For this purpose, several authors dedicated efforts to the modification of the 3D printed surface to incorporate smart materials or specific ligands to improve selectivity [6, 23–25], yet little efforts geared towards the decoration of the surface with bioselective ligands, such as antibodies (Abs). The use of natural Abs immobilized on a solid support (immunosorbents, ISs) is an appealing alternative to standard SPE materials because of the high selectivity and affinity inherent to the antigen–antibody interactions. To date, Abs have been combined with 3D printed devices for the improvement of ELISA methodologies [26, 27], but their combination with immunoaffinity extraction has been scarcely studied. For example, Parker et al*.* [28] immobilized Abs by physical adsorption onto a polymer monolith, but without Ab covalent attachment. To the best of our knowledge, the combination of 3D printed devices with IS-based stirring sorptive (micro)extraction (containing covalent attached Abs) has not been studied yet. In this context, the current work presents the design and optimization of tailor-made cup-shaped 3D printed stirring scaffolds for immunoaffinity-based SPE aimed at high preconcentration factors by using high sample loading amounts and low elution volumes. The as-prepared 3D printed devices will be decorated by a monoclonal Ab (mAb) against diclofenac (DCF) to isolate the target species from wastewaters before its subsequent elution and determination by HPLC–UV. For this purpose, different procedures to covalently attach the mAb onto the 3D printed surface and the extraction conditions (time, temperature, spinning speed and elution mode and buffer) involving amidation reactions or Schiff base formation will be thoroughly studied to enable maximum extraction capacity for DCF. Other aspects such as non-selective interactions of the pristine 3D printed surface will be also evaluated.

## Experimental

Detailed information of (i) reagents, standard solutions and samples (influent wastewaters) and (ii) chromatographic system and other instrumentation is available as Supporting Information (SI). The fabrication of the 3D printed devices, the immobilization of the mAb and the SBSE protocol are described below.

### Design and fabrication of the spinning cup-based 3D printed device

**Fig. 1:**
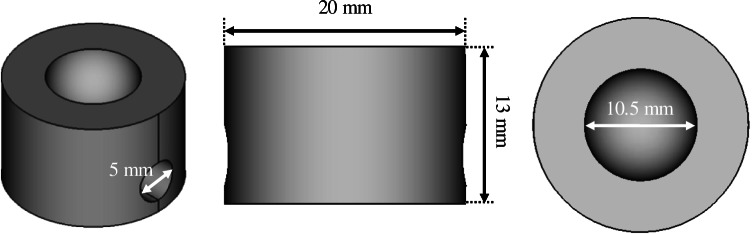
CAD design of the 3D printed SC3D device including the dimensions of the main components

The spinning cup-shaped 3D (SC3D)-printed device was designed using the software FreeCAD® 0.18 (www.freecadweb.org). A diagrammatic illustration of the SC3D platform is shown in Fig. 1. To this end, a cylindrical rod of 20 × 13 mm (d × h) was initially designed to which a semi-sphere of 10.5 mm of diameter was removed from the upper face. Hence, a cup-shaped cavity with a nominal volume of 300 µL was obtained. In addition, an empty cylinder of 5 × 20 mm (d × h) was modelled under the cup-shaped cavity (see Fig. 1) in order to incorporate a magnet for the immunoaffinity stirring sorptive extraction (ISSE) protocol. The computed-aided design (CAD) of the 3D printed device in stereolithography (STL) format can be found in Supporting Information.

The SC3D model was loaded in the computer-aided manufacturing (CAM) software (Preform, Formlabs, Somerville, USA) for positioning and slicing. We selected adaptable resolution, that is, the software decides upon what zones are in need of high resolution (25 µm) and those of less resolution (100 µm), including intermediate resolutions, depending on the design and position. The object was placed with the cavity facing downwards in order to avoid unnecessary supports onto the cavity. Minisupports were however included in the printing CAM software for the base of the device (contact point of 0.4 mm and 3 mm height). The final disposition of the SC3D design was transferred to the SLA Form 3 printer (Formlabs) for printing up to 36 SC3D pieces at a time using the FLGPCL04 (Formlabs) clear resin (238 layers and 3.82 mL/piece).

After the printing process, the green-state SC3D prints were removed from the moving platform and soaked in isopropanol (IPA). The supports were removed from the prints using a cutter or by hand whenever possible. In order to eliminate the non-polymerized resin from all the parts of the print, the SC3D devices were cleaned by bath sonication using (i) IPA, (ii) deionized water and (iii) IPA again for 15 min each step. Then, the objects were dried under a N_2_ stream and finally were exposed to UV for 1 h (2 × 16 W low-pressure mercury lamps) in an UV oven (KA-9180, PSKY, China).

### Modification of the upper cavity of the spinning cup with monoclonal antibody

**Fig. 2:**
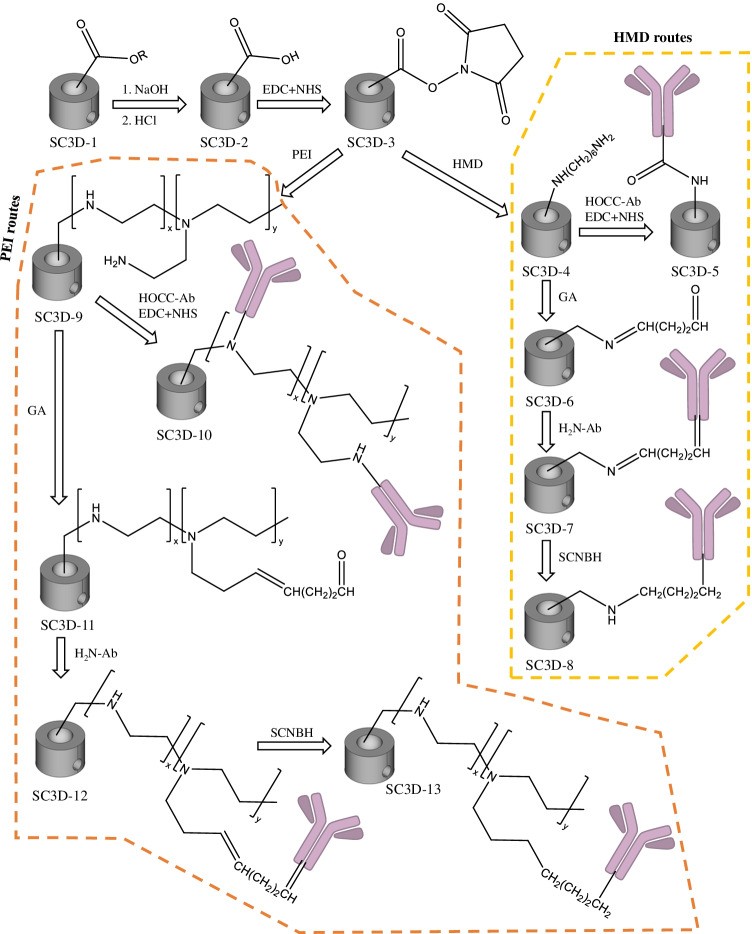
Scheme of the different reaction pathways for the covalent immobilization of mAb against DCF onto the SC3D devices. Only the main chemical moieties involved in the covalent reactions are illustrated for the sake of simplicity and readability. The reaction conditions used for each step are indicated in Experimental

Various immobilization protocols were assessed for covalent attachment of the monoclonal antibody against diclofenac (mAb_DCF_) onto the SC3D platform. In all the steps, only the upper cavity of the SC3D device was functionalized. Therefore, the derivatization solutions were merely placed in the cup-shaped cavity (*ca.* 300 µL). The protocol is based on a previous publication [6] with some modifications to attach the mAb_DCF_ to the walls of the printed device (see Fig. 2). Briefly, the as-cleaned and cured 3D printed device (SC3D-1, see Fig. 2) was allowed to react with NaOH (SC3D-2) to transform the ester group from the polymerized resin into a carboxylic group (4 h at 60 ºC with 2 M NaOH in water). The SC3D-2 device was then subjected to an aqueous solution containing 0.2 M 1-ethyl-3-(3-dimethylaminopropyl)carbodiimide (EDC) and 0.3 M N-hydroxysuccinimide (NHS) for 30 min at 60 ºC to obtain the SC3D-3 device. From this point, two strategies were followed: (i) modification with 0.5 M hexamethylenediamine (HMD) solution in water for 1 h at 60 ºC (SC3D-4) and (ii) modification with 0.5 M polyethyleneimine (PEI) in water for 1 h at 60 ºC (SC3D-9). The incorporation of the antibody was performed by two different strategies for both 3D printed devices, SC3D-4 and SC3D-9. The first protocol was based on the direct attachment of the mAb_DCF_ (0.1 mg mL^–1^) through its free COOH moieties by using 0.2 M EDC and 0.3 M NHS as coupling agents (15 h at R.T.) to obtain SC3D-5 (from i) and SC3D-10 (from ii). The second approach consisted of the covalent immobilization of the mAb_DCF_ through its free NH_2_ moieties. For this purpose, preliminary devices, SC3D-6 (from i) and SC3D-11 (from ii), were obtained by reaction of HMD or PEI, respectively, with a 50% glutaraldehyde (GA) aqueous solution (12 h at R.T.) [29]. Then, the mAb_DCF_ was attached to both devices by adding 300 µL of a solution containing 0.1 mg mL^–1^ of mAb for 12 h at R.T., thus obtaining SC3D-7 (from i) and SC3D-12 (from ii). Finally, a water solution of 0.2 M of sodium cyanoborohydride (SCNBH) solution was added to SC3D-7 and SC3D-12 at 4 ºC for 2 h [29] in order to reduce the double bounds of the chains, thus yielding the final SC3D-8 (from i) and SC3D-13 (from ii) mAb-laden prints.

### Immunoaffinity stirring sorptive extraction protocol

The ISSE procedure for the extraction of DCF from aqueous samples and influent wastewaters (see Fig. S1) using the optimal SC3D derivatization procedure (see Results and Discussion section) is described as follows: first, the mAb-containing SC3D device is stirred for 30 min at 300 rpm in 50 mL of an unfiltered wastewater sample (or standard) in 10 mM phosphate saline solution (PBS) at pH 7.4 and 35 ºC (see Video S1). Then, the SC3D device is retrieved from the sample and dried with an N_2_ stream. A stagnant washing step is performed by adding 300 µL of 10 mM PBS to the upper cavity for 10 min with the subsequent removal of the solution and drying of the cavity again by N_2_. The elution step is performed with 300 µL of an aqueous solution containing 0.1 M of glycine (Gly) and 1% of sodium dodecyl sulphate (SDS) at pH 10. The elution solvent is added to the upper cavity, and DCF retrieval is assisted by ultrasound irradiation for 20 min (see Video S2). Then, 100 µL of the eluate is collected and transferred to an HPLC vial to which 50 µL of a 30 mM PBS solution is added prior to its analysis by HPLC–UV (see Supporting Information for the HPLC conditions).

## Results and Discussion

### Chemical characterization of the 3D printed spinning cup-shaped immunosorptive device

The covalent binding of properly oriented mAb_DCF_ is a crucial step to enable high immunoextraction capacity of the SC3D systems. For this purpose, four different strategies to covalently attach the mAb_DCF_ have been herein assessed (see Fig. 2 and Experimental). Briefly, after amination of the 3D printed surface (HMD with primary amines and PEI with a mixture of primary, secondary and tertiary amines) by the EDC/NHS coupling reaction, two different approaches were used in either case: (i) direct incorporation of the mAb_DCF_ by the EDC/NHS coupling reaction to the HMD (SC3D-5) and PEI (SC3D-10) residues and (ii) modification with GA followed by decoration with the mAb_DCF_ (SC3D-7 and SC3D-12, respectively) via Schiff base formation and a final reduction of the residual double bonds to single bonds with SCNBH (SC3D-8 and SC3D-13, respectively). Quantitation of the amount of mAb_DCF_ attached onto the surface of the SC3D-5, SC3D-8, SC3D-10 and SC3D-13 devices was performed via EDAX analysis of %S onto a 10 µm^2^ of SC3D cup surface. As shown in Fig. S2, the highest amount of S (and therefore of mAb_DCF_) was found in the SC3D-8 device (0.22% S, wt.). The low amount of mAb_DCF_ attached to SC3D-5 (0.01% S, wt.) compared to SC3D-8 can be explained by the fact that the EDC/NHS coupling reaction is a zero-length spacer arm in contrast to Schiff base GA-based reactions and, thus, the steric hindrance to the direct coupling of mAb_DCF_ to HDM (SC3D-5) would lead to a less favourable attachment. In the case of SC3D-10 (0.06% S, wt.), the lower content of mAb_DCF_ compared to SC3D-8 can be likewise explained by the steric hindrance of the PEI to the covalent attachment of mAb_DCF_ via EDC/NHS coupling despite having more available binding sites for mAb. In fact, the larger amount of primary and secondary amines from PEI in SC3D-10 vs SC3D-5 (by direct HMD attachment) led to a sixfold mAb_DCF_ increase. The incorporation of a longer spacer arm to PEI using GA (SC3D-13), however, did not ameliorate the yield (0.02% S, wt.) compared to SC3D-10. This result can be attributed to the limited amount of functional groups available for reaction with GA since only primary amines can react with GA, thereby avoiding the potential anchorage of mAb_DCF_ to the secondary amines like in SC3D-10. Notwithstanding the results presented in Fig. S2, the four synthetic strategies will be tested in the following studies to assure that the various reactions did not change the quaternary structure of the antibody nor alter the paratope, thus keeping the affinity interactions with DCF.

The morphology of the 3D printed upper cavity has been examined by scanning electron microscopy (SEM) for both the SC3D-1 and SC3D-8 as shown in Fig. S3. The SEM monographs showed a significantly different surface morphology for SC3D-8 that serves as an indirect measurement of the success of the covalent derivatization reactions.

### Investigation of the sorptive potential of the 3D printed immunoaffinity materials

**Fig. 3:**
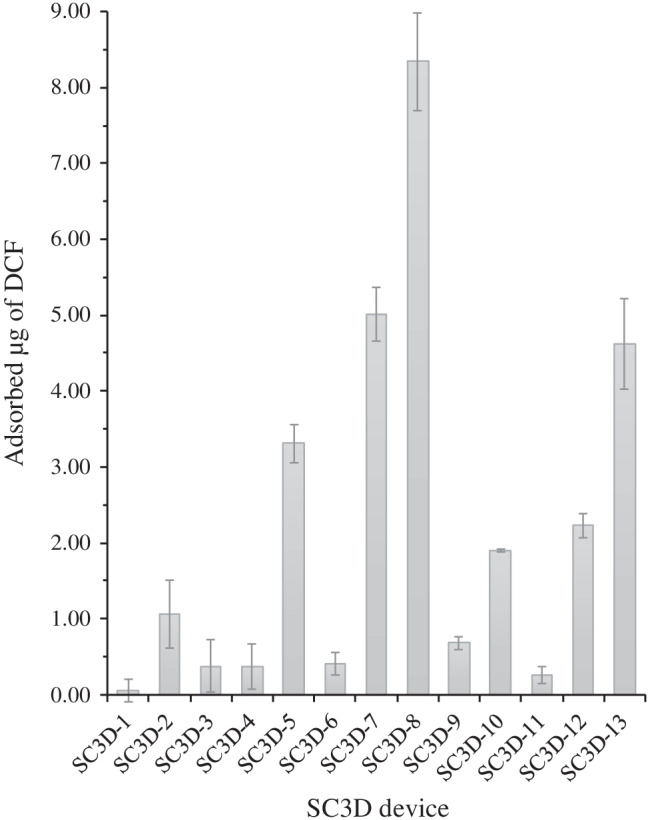
Adsorbed amount of DCF from a solution containing 50 mL of 1 mg L^–1^ DCF (10 mM PBS; 300 rpm; 30 min; 25 ºC) for all the 3D printed devices (see Fig. 2). All the experiments were performed in triplicate except for SC3D-8 which was performed in sextuplicate

The loading capacity of all of the prepared 3D printed SC3D devices (SC3D-1 to SC3D-13) was evaluated by measuring their adsorption capacity against 1 mg L^–1^ of DCF in 10 mM PBS. Figure 3 shows the amount of DCF adsorbed (including standard deviation) onto every 3D printed spinning cup-shaped platform.

As it can be observed, the non-specific interaction of the 3D printed spinning device is negligible (SC3D-1, 0.06 µg of DCF). In addition, all the other reaction steps excluding mAb did not show significant adsorption capacity (up to 1.0 µg of DCF for SC3D-2). In any case, the incorporation of the mAb_DCF_ increased the adsorption capacity by 64 to 95% as compared to the preceding reaction step. This behaviour can be explained by the selection of an adequate pH (pH > pKa_(DCF)_ + 1) that restricts the non-selective reversed-phase extraction of the non-modified walls of the device, thereby promoting the capture of DCF by the mAb_DCF_. Nevertheless, the differences in the amount of mAb covalently attached to the surface for the HMD protocols (SC3D-5 and SC3D-8) are not comparable with their respective adsorption capacities. For example, the SC3D-5, despite the low amount of S on the surface (0.01% S, wt.), features a reasonable adsorption capacity (40% of that obtained with the SC3D-8). To explain this observation, it should be born in mind that the mAb_DCF_ is incorporated through the COOH moieties in SC3D-5, in contrast to SC3D-8 for which the attachment is performed through the NH_2_ moieties of mAb_DCF_. Therefore, the availability of the paratope that contains free terminal amino moieties for interaction with DCF might be partially jeopardized in SC3D-8. In case of PEI procedures, notwithstanding the large amount of mAb_DCF_ decorating the SC3D-10, a better adsorption capacity is obtained by the SC3D-13. This finding suggests that the paratope structure and availability of the N-terminus are not hindered in SC3D-13 by the covalent attachment of the mAb. Also, the branched structure of PEI in SC3D-10 does not make DCF readily accessible to the binding sites of mAbs linked to secondary amines. By comparing SC3D-8 versus SC3D-13, the spatial disposition of the mAb_DCF_ onto the surface seems to be more appropriate for SC3D-13, but this is offset by the enhanced loading of mAb_DCF_ onto SC3D-8. The reduction of the double bonds (SC3D-7 to SC3D-8 and SC3D-12 to SC3D-13) of the GA crosslinking step is proven to ameliorate the adsorption capacity significantly (increase by 40 and 52% for the HMD and PEI protocols, respectively). Probably, the free rotation of the single bonds gives greater adaptability to the mAb_DCF_-containing structure to facilitate access of DCF to the paratope. Therefore, the SC3D-8 device was selected for further studies and application to real sample analysis.

### Analytical performance of the DCF sorptive immunoaffinity extraction protocol

The ISSE protocol using the novel 3D printed spinning platforms has been optimized by evaluating the effect of several experimental parameters, such as the use of bovine serum albumin (BSA) as a capping agent, sample loading time, reaction temperature, stirring speed, elution mode and elution solvent on the preconcentration and retrieval of DCF. Firstly, the use of BSA as a capping agent to block the non-selective interactions of the 3D printed material was evaluated. To this end, the entire SC3D-8 device was soaked in 1 mg mL^–1^ BSA containing 10 mM PBS for 2 h. Then, the platform was cleaned with 10 mM PBS and dried under a N_2_ steam pending use. The adsorption capacity of the SC3D-8 device in the absence of BSA was 8.3 ± 0.6 µg of DCF as compared to 8.7 ± 0.9 µg of DCF for the counterpart capped with BSA (see Supporting Information for the capping procedure). A *t* test of comparison of means revealed the inexistence of statistically significant differences on the sorptive capacity of both devices (*t*_exp_ = 0.693 at α = 0.05 for three degrees of freedom, *t*_*crit*_ = 3.182), and therefore, no capping with BSA was used in the following studies. Three different parameters related to the loading step were investigated in detail: time (Fig. S4A), temperature (Fig. S4B) and stirring speed (Fig. S4C). As for the extraction time, steady-state extraction conditions were observed from 30 min onwards. In the case of temperature, the mAb_DCF_ shows superior performance at temperatures (35 ºC) close to those of physiological conditions in mammals (37 ºC). At 35 ºC, a twofold increase of the adsorbed DCF (19.4 ± 0.7 µg of DCF) against room temperature (8.3 ± 0.6 µg of DCF) was observed. The adsorption capacity was also studied at different agitation rates of the SC3D device at the loading step (300–1000 rpm). It should be noted that non-homogeneous stirring was observed down to 300 rpm because of the friction of the polymeric device with the walls of the beaker. However, increasing the stirring speed above 300 rpm led to a sharp decrease of the adsorption capacity (Fig. S4C). The vortex generated in the body of the solution most likely jeopardized the DCF diffusion towards the paratope within the cavity of the stirring device. Therefore, the following extraction conditions were selected for further studies: 30 min, 35 ºC and 300 rpm.

A critical step related to the use of immunosorbents as sorptive materials is the elution step. In our work, the DCF elution was assessed in terms of elution mode and buffer composition (see Table 1). Firstly, 9 different organic buffer solutions as indicated in Table 1 were tested in the static mode, that is, the SC3D-8 cavity was fully filled with 300 µL of buffer, and the eluate was taken out after 10 min followed by HPLC analysis. However, DCF was detected chromatographically in none of the eluates. Hence, ultrasound (US)-assisted elution using the same 9 buffers was tested instead. Nevertheless, US bath sonication might lead to losses of eluent or the addition of water from the bath into the upper cavity. Therefore, the amount of aqueous elution solution existing in the SC3D-8 device after 30 min of US irradiation was estimated. For this purpose, *ca.* 300 mg of water was placed into the upper cavity and keep for 30 min in the US bath. The remaining volume that was collected and weighed (299.1 ± 0.3 mg, n = 3) indicated that no significant losses occurred (paired *t* test with a *t*_*exp*_ = 3.327 at α = 0.05 for two degrees of freedom, *t*_crit_ = 4.303). Then, the different buffers were tested under US irradiation for DCF elution for a preset time of 10 min (see Table 1). Nevertheless, the absolute recoveries were in all instances below 52% except for the buffer 9, based on a mixture of Gly and SDS in water at pH 10 that yielded a *ca.* 62% of absolute recovery. Therefore, the elution time was increased to 20 and 30 min exploiting buffer 9 (buffers 10 and 11, respectively). As can be seen from Table 1, absolute recoveries as high as 96% were obtained, without statistically significant differences between 20 and 30 min. Therefore, an elution time of 20 min using 0.1 M Gly + 1% SDS at pH 10 was selected for further studies.

**Table 1:** Absolute DCF recoveries obtained for various elution solutions under US irradiation as applied to the SC3D-8 device

Buffer	Solvent	Additives	pH	Time (min)	Absolute recovery (%)
1	ACN	-	-	10	40.4 ± 0.6
2	ACN	2% FA	-	10	45.1 ± 0.2
3	H_2_O	2% FA	2.3	10	51.8 ± 0.7
4	H_2_O	0.1 M Gly-HCl	2.0	10	4.8 ± 0.6
5	H_2_O	0.1 M Gly-NaOH	2.0	10	19.4 ± 0.4
6	H_2_O	20 mM MES + 3.5 M MgCl_2_	6.5	10	15.8 ± 0.3
7	H_2_O	8 mg L^–1^ 2-ME + 2% SDS + 62.5 µM Tris	6.8	10	20.9 ± 0.6
8	H_2_O	6 M Urea	-	10	12.8 ± 0.5
9	H_2_O	0.1 M Gly + 1% SDS	10.0	10	61.5 ± 0.8
10	H_2_O	0.1 M Gly + 1% SDS	10.0	20	96.3 ± 0.8
11	H_2_O	0.1 M Gly + 1% SDS	10.0	30	94.8 ± 0.7

*Abbreviations: formic acid (FA); glycine (Gly); sodium dodecyl sulphate (SDS); 2-(N-morpholino)ethanesulfonic acid (MES); 2-mercaptoethanol (2-ME)*

After investigation of the loading and elution conditions of the ISSE protocol, analytical parameters such as the loading capacity and the breakthrough volume were measured. The maximum loading capacity of SC3D-8 was found to be 19.4 ± 0.7 µg DCF (n = 3). The breakthrough volume was evaluated between 10 and 500 mL of a standard containing 0.5 µg of DCF, and excellent absolute recoveries were found for volumes spanning from 10 to 50 mL (95.5–95.7%). However, the absolute recoveries decreased to 56.6 and 44.1%, whenever the volumes increased to 100 and 500 mL, respectively. Therefore, the sample volume was fixed to 50 mL for evaluation of the analytical performance and application to real samples.

The limit of detection (LOD) and limit of quantification (LOQ) were estimated based on a signal-to-noise ratio of 3 and 10, respectively. The LOD and LOQ were 108 and 360 ng DCF L^–1^, respectively. A good linearity (R > 0.998) was obtained within the concentration range of 0.4–1,500 µg L^–1^ with a sensitivity (slope of the calibration curve) of 30.7 mV L µg^–1^. The device-to-device reproducibility for SC3D-8 device was excellent (RSD: 3.8%, n = 6). The maximum enrichment factor for a 50-mL sample volume was estimated as 159 (after dilution with PBS was 106) for standard solutions and 125 (after dilution with PBS was 83) for influent wastewater (see section below). Another interesting advantage of the ISSE protocol is the moderately short extraction/elution times for nearly quantitative retrieval of DCF (< 1 h), which in combination with the possibility of performing several procedures simultaneously facilitate its use in routine laboratories. In terms of reusability, the SC3D-8 devices can be stored at 4ºC for more than one week without compromising the absolute recoveries, although the devices can be used only once because a decrease of 13% of the absolute recovery was found after the first use followed by a drastic lessening down to 85% after the second extraction. However, the beauty of SLA 3D printing as a rapid and cost-effective prototyping technique could be herein fully leveraged inasmuch as up to 36 stirring devices were printed in a single run. The estimated cost is less than 0.7 € per print, yet increased up to 16 €/device with the covalent immobilization of the mAb. However, the price can be significantly reduced whenever mass production is intended for the 3D printed platforms.

### Application of the optimized ISSE protocol for the extraction of DCF in influent wastewaters

The 3D printed SC3D-8 device with covalently immobilized mAb_DCF_ was applied to the extraction and preconcentration of DCF in raw (influent) wastewaters. For this purpose, two analyte-free 24-h composite wastewaters from Palma de Mallorca, Spain, (EDAR-I (wastewater 2) and EDAR-II (wastewater 1) (see Supporting Information), were spiked at the 2 and 10 µg DCF L^–1^ levels based on previous literature [30]. The absolute recoveries calculated against the external mass calibration using standards in 10 mM PBS ranged from 76 ± 3 to 80 ± 5% for wastewater 1 and 79 ± 2 to 80 ± 5% for wastewater 2. The results indicated statistically significant differences as compared to the absolute recoveries obtained with standard solutions (96.3 ± 0.8%) as indicative of matrix effects. In any case, there were no statistically significant differences in the absolute recoveries between the two spike levels and between the two wastewater samples (*t*_exp_ = 0.623 at α = 0.05 for 10 degrees of freedom, *t*_*crit*_ = 2.262). Hence, the relative recoveries for the two spike levels and wastewater samples were calculated using an external calibration but corrected by the average absolute recovery in wastewaters (78.6%). Thus, the relative recoveries in wastewaters for the two spike levels ranged from 102 ± 2 to 109 ± 6%. The chromatograms of the direct injection of a PBS blank, unprocessed EDAR-II wastewater doped with 10 µg L^–1^ of DCF and the EDAR-II wastewater doped with 10 µg L^–1^ of DCF after the ISSE-CS3D-8 protocol are illustrated in Figs. S5A, S5B and S5C, respectively.

### Comparison with other rotating device-based sorptive extraction procedures

Table 2 comprehensively compares the analytical parameters of the ISSE protocol developed in this work using 3D printed SC3D devices with those of previous rotating disk-based extraction methods for determination of DCF in wastewaters and biological samples. The LOD of our protocol is similar to those reported in the literature, even of those methods using mass spectrometric detection, but after application of an evaporation and reconstitution step. Since our methodology avoids eluate evaporation, lower LODs are actually achieved in this work compared with those calculated without the evaporation step (see Table 2). Also, non-exhaustive extraction is usually reported for previous microextraction techniques exploiting stirring devices [8, 9, 31]. In summary, the combination of the satisfactory analytical figures of merit along with the unrivalled enrichment factor of the ISSE protocol without eluate processing makes this methodology suitable for the determination of DCF in influent wastewaters at the expected concentration levels.

**Table 2:** Overview of the analytical performance of recently reported rotating-based sample preparation methods prior to chromatographic separation and determination of DCF in wastewaters and biological samples

Material	Sample	Method	LOD^1^ (ng L^–1^)	Linearity (µg L^–1^)	E.R.^3^	Absolute recovery (%)	RSD (%)	Reference
Octadecyl-modified silica	Urine	SI-based RDSE-LC-UV	2,600 (217)	200–2,000	1.5–2.3	55 (standard) 38 (sample)	3.2	[8]
MIP	Wastewaters	RDSE-GC–MS	1,200 (67)	-	5	50 (standard)	5.0	[9]
Oasis-HLB	Wastewaters	RDSE-GC–MS	3,300 (40)	0.1–1,000	2	33 (standard)	20	[31]
Oasis-HLB	Wastewaters	RDSE-LC–MS	890 (89)	10–1,000	5	95 (standard)	5.9	[32]
EDA-GMA-based monolith	River waters	SBSE-LC–MS	225 (75)	0.1–100	3.3	63 (standard) 47 (sample)	< 9	[33]
3D printed immunosorbent	Wastewaters	ISSE-LC-UV	106^2^	0.4–1,500	> 87^2^	97 (standard) 80 (sample)	3.8	This work

^1^ LOD estimated before the evaporation step and between brackets those obtained after eluate evaporation

^2^ LOD obtained without eluate evaporation

^3^ E.R. before the evaporation step

Abbreviations: E.R. (enrichment factor); MIP (molecularly imprinted polymer); RDSE (rotating disk sorptive extraction); GC (gas chromatography); MS (mass spectrometry); SI (sequential injection); LC (liquid chromatography); EDA (ethylenediamine); GMA (glycidyl methacrylate)

## Conclusions

In this work, a 3D printed spinning cup-shaped extraction device covalently incorporating an mAb against DCF has been developed for retrieval of the target analyte from influent wastewaters followed by HPLC–UV analysis. This paper demonstrates for the first time the feasibility of 3D printing to fabricate devoted and midget designs for sample preparation by derivatization of selected regions of the printed surface with bioselective ligands to improve the adsorption and preconcentration capacity of the 3D printed devices in sorptive extraction and stirring integrated methodologies. In fact, the printing of the SC3D device can be performed really fast (36 platforms in 8 h) at low cost (0.68 €/print). The overall cost is augmented to 16 €/device by incorporating the mAb, but still deemed affordable for the fabrication of a selective sorptive device for processing untreated influent wastewaters.

The covalent attachment of the mAb_DCF_ onto the 3D print has been comprehensively studied by evaluating four synthetic pathways for efficient extraction of DCF in wastewaters. The amount of attached antibody was elucidated by the %S onto the 3D printed surface as obtained by EDAX analysis. The potential non-selective interactions from the photopolymerized SLA material were evaluated, and it was demonstrated that the DCF extraction by untreated devices (without Abs) is negligible. Using the optimal modified device, different experimental parameters (loading time, extraction temperature, stirring speed, and the elution mode and elution solution) were investigated. The as-prepared spinning-cup device proved to be rugged and reliable for the extraction and preconcentration of DCF from unfiltered wastewaters with excellent analytical performance. The main shortcoming of the SC3D device relates to its reusability (only once). However, the possibility of obtaining high enrichment factors without solvent evaporation and reconstitution steps enables highly versatile microscale extraction methodologies for coupling to HPLC.

As far as we are concerned, the combination of selective elements and/or advanced (nano)structured materials with 3D printed scaffolds in stirring-enabled sorptive extraction systems is a novel approach that is expected to open new avenues in the development of dedicated devices for specific applications in the environmental and bioanalytical fields.

## Supplementary Information

Below is the link to the electronic supplementary material.

Supplementary file1 (DOCX 7039 KB)

Supplementary file2 (STL 72 KB)

Supplementary file3 (MP4 1737 KB)

Supplementary file4 (MP4 4045 KB)

## Data Availability

Relevant data generated and analysed during the current study including the FreeCAD design of the SC3D device are available in Zenodo repository (DOI: 10.5281/zenodo.6397011).
