# Supplementary material for: 3D printed spinning cup-shaped device for immunoaffinity solid-phase extraction of diclofenac in wastewaters
Source: Mikrochim Acta. 2022 Apr 2;189(5):173. doi: 10.1007/s00604-022-05267-9 (PMC8976768; doi:10.1007/s00604-022-05267-9)
Supplement: Supplementary file 1 — Supplementary file1 (DOCX 7039 KB) [file 604_2022_5267_MOESM1_ESM.docx]

**SUPPORTING INFORMATION**

**3D printed spinning cup-shaped device for immunoaffinity extraction of diclofenac in wastewaters**

Enrique Javier Carrasco-Correa^1^*, José Manuel Herrero-Martínez^1^, Ernesto Francisco Simó-Alfonso^1^, Dietmar Knopp^2^, Manuel Miró^3^*

*^1^ University of Valencia, Spain, Department of Analytical Chemistry, University of Valencia, C/Doctor Moliner 50, 46100 Burjassot Valencia*

*^2^ Department of Chemistry, Technical University of Munich, Elisabeth-Winterhalter Str. 6, 83177 München, Germany*

*^3^ FI-TRACE group, Department of Chemistry, University of Balearic Islands, Carretera de Valldemossa, km 7.5, E-07122 Palma de Mallorca, Spain*

**9 pages**

**5 figures**

*Corresponding authors:

**Dr. Enrique Javier Carrasco-Correa**
e-mail: enrique.carrasco@uv.es
Tel.: +34963544248
Fax: +34963544436

**Prof. Manuel Miró**
e-mail: manuel.miro@uib.es
Tel: +34 971172746
Fax: +34 971173426

1. **Experimental Section**
   1. **Reagents, standards, and samples**

All reagents were of at least analytical reagent grade. Highly pure deionized water was obtained from a Milli-Q system (resistivity > 18.2 MΩ·cm, Merck-Millipore, Molsheim, France). Stock solutions of diclofenac (DCF) were prepared from a solid analytical reagent grade (Fisher Scientific, Walthan, USA) in methanol (MeOH), and were stepwise diluted in water for further assays. 2-propanol (ACS Basic, Scharlau, Barcelona, Spain) was used for cleaning of the 3D stereolithographic prints. HPLC-grade ACN, MeOH and acetic acid (HAcO) were from Scharlau. Hexamethylenediamine (HMD), sodium cyanoborohydride, 1-ethyl-3-(3-dimethylaminopropyl)carbodiimide (EDC), N-hydroxysuccinimide (NHS), polyethyleneimine, branched (1800 kDa, PEI), glutaraldehyde (GA), glycine (Gly), sodium dodecyl sulphate (SDS), magnesium chloride, sodium hydroxide, hydrochloric acid, urea and tris(hydroxymethyl)aminomethane were obtained from Fisher Scientific. Formic acid, bovine serum albumin (BSA) and 2-mercaptoethanol were acquired from Merck KGaA (Darmstadt, Germany).

Two influent (raw) wastewaters from distinct wastewater treatment plants (WWTP) in Palma de Mallorca (Illes Balears, Spain) were analyzed in this work. 24-h composite wastewaters were obtained automatically by collecting 100 mL-wastewater every 15 min. After sampling, 1L-composite sample was immediately frozen at -20 ºC without any sample treatment. Prior to analysis, the samples were kept at 4ºC for 6 h and afterwards, 0.5 mL of 1 M phosphate buffer saline (PBS) was added to a final buffer concentration of 10 mM. Unfiltered samples were analyzed. Wastewater 1 was sampled from WWTP EDAR-II that serves 480,000-population equivalents from Palma city and boroughs (namely, Bunyola, Esporles and Marratxi). Wastewater 2 was sampled from WWTP EDAR-I that serves ca. 410,000- population equivalents, mainly from coastal areas (Palma Beach and Lluchmajor neighborhoods). The chemical composition of wastewater 1 was as follows: pH =7.52; [N]_total_= 62 mg L^-1^; [P]_total_=10.1 mg L^-1^; COD= 866 mg L^-1^ O_2_ and BOD_5_= 540 mg L^-1^ O_2_. Wastewater 2 was composed of pH= 7.43; [N]_total_= 75 mg L^-1^; [P]_total_=18.6 mg L^-1^; COD= 1115 mg L^-1^ O_2_ and BOD_5_= 500 mg L^-1^ O_2._

- 1. **Antibody isolation and purification**

The high affine (K_D_ of 1.5 ×10^-10^ M) mouse monoclonal anti-diclofenac antibody mAb 12G5 (subtype IgG2b, к) was affinity purified from hybridoma cell culture supernatant (CCS) using goat anti-mouse IgG (GAM) immunoaffinity chromatography. A buffered stock solution of 5 mg mL^-1^ mAb was prepared in 50 mM Na_2_HPO_4_, 20 mM NaH_2_PO_4_, Tris-HCl 0.1 M, pH 7.4, azide 0.02% and kept in the fridge until use.

- 1. **Instrumentation**

A high-performance liquid chromatographic (HPLC) module system (Jasco, Tokyo, Japan) controlled by the ChromNAV 2.0 software was used throughout as analytical separation apparatus. The chromatographic setup integrates (i) a PU-4180 RHPLC pump that endures pressures up to 700 bar, (ii) an AS-4050 HPLC autosampler equipped with a high pressure 6-port rotary injection valve furnished with 1/32” i.d. PEEK loop of 50 µL, (iii) a GECKO 2000 column heater, (iv) an MD-4017 photodiode array detector, and (v) an Onyx monolithic HD-C18 analytical column (100 × 4.6 mm, Phenomenex, Torrance, CA, USA) preceded by a security guard column Onyx monolithic C18 guard cartridge (5 × 3 mm, Phenomenex). The HPLC system was operated under isocratic mode using a mobile phase consisting of 50/50 (v/v) ACN/H_2_O mixture containing 0.1% (v/v) formic acid. The flow rate was 1 mL min^–1^, the column temperature was kept to 30 ºC, and the injection volume was in all cases 20 μL. The analyte was measured at a detection wavelength of 276 nm. After direct analysis of the unprocessed wastewater (Fig. S3B) three water injections of 50 µL each followed by cleaning the analytical column with mobile phase for 1 h at 1 mL min^–1^ was performed so as to avoid cross-contamination effects. In addition, a DCF standard injection at the 500 µg L^-1^ level was done as QC to confirm that the chromatographic parameters for DCF remained unaltered after the analysis of the raw wastewater.

Scanning electronic microscopy (SEM) images and energy dispersive analysis by X-ray (EDAX) of materials were performed by a scanning electron microscope (S-4800, Hitachi, Ibaraki, Japan) provided by a field emission gun and an EMIP 3.0 image data acquisition system (Rontec, Normanton, UK, www.rontec.com). All samples for SEM analysis were previously sputtered-coated with Au/Pd for 2 min to avoid charging issues.

- 1. **Calculation of absolute and relative recoveries and enrichment factors**

The formula of the absolute and relative recoveries and enrichment factor is given as follows:

$Absolute Recovery (AR,\%)=\frac{mass {DCF}_{eluted}}{mass {DCF}_{loaded}}\times100$

$Relative Recovery (RR, \%)=\frac{{[DCF]}_{found}}{[{DCF]}_{spiked}}\times100$

*Enrichment Factor (EF)* = $\frac{AR}{100}$ ×$\frac{Vsample}{(Veluate+Vbuffer)}$

where DCF_eluted_ is the DCF mass which has been retrieved in the elution step and analyzed by HPLC; DCF_loaded_ is the mass of DCF that is available to be extracted in the starting solution; and [DCF]_found_ in the sample is calculated as follows:

[DCF]_found_ = $\frac{\left[ DCF \right]eluate}{EF}$

Fig. S1. Scheme of the ISSE procedure for the extraction of DCF from standards and samples using the mAb-laden SC3D device.

**Fig. S2.** Percentage of S, related to the attached antibody against DCF in a 10 µm^2^ section of the four final mAb_DCF_-laden SC3D devices.

**Fig. S3 –** SEM micrographs (magnification: ×15k) of the pristine 3D printed SC3D-1 platform (A) and the SC3D-8, which was modified with the monoclonal antibody against DCF (B).

**Fig. S4 –** Adsorbed amount of DCF in a single SC3D-8 device from a solution containing 1 mg L^–1^ of DCF in 10 mM PBS using various sample loading conditions: time (A), temperature (B) and stirring rate (C). Other conditions: 25 ºC and 300 rpm (time, A); 30 min and 300 rpm (temperature, B); 30 min and 35 ºC (stirring speed, C).

**Fig. S5 -** Chromatograms obtained for a PBS blank (A), direct injection of an influent wastewater containing 10 µg of DCF L^–1^ (B) and the same sample containing 10 µg of DCF L^–1^ after the ISSE extraction protocol using the 3D-printed mAb_DCF_-laden SC3D-8 device (C). Chromatographic conditions are described in section 1.3 above.
